# Grand Challenges in Consciousness Research Across Perception, Cognition, Self, and Emotion

**DOI:** 10.3389/fpsyg.2021.770360

**Published:** 2021-11-26

**Authors:** Antonino Raffone

**Affiliations:** ^1^Department of Psychology, Sapienza University of Rome, Rome, Italy; ^2^School of Buddhist Studies, Philosophy and Comparative Religions, Nalanda University, Rajgir, India

**Keywords:** consciousness, perception, cognition, self, emotion, dreaming, mindfulness

## Introduction

The field of consciousness research has developed over the last decades by interdisciplinary and multi-method investigations, through the integration of theoretical, empirical and clinical studies. Consciousness research is relevant across philosophy, psychology, neurology, cognitive and affective neurosciences, cognitive science, and artificial intelligence, among other disciplines. The implicated research methods include psychophysical, psychophysiological and behavioral methods, subjective reporting (with or without trained introspection), electrophysiology and neuroimaging, the brain lesion method, and computational modeling. Consciousness has also been studied in developmental, lifespan, comparative, and evolutionary perspectives. Insights have also been gained by deficient consciousness (e.g., due to brain lesions) and enhanced awareness (e.g., related to meditation states and traits) (e.g., Lutz et al., 2008; Banks, 2009; Bayne et al., 2009; Seth, 2010; Gosseries et al., 2014).

This article will highlight some current grand challenges in consciousness research. Several core problems in consciousness research historically implicate a tension between opposite views. These include the antithetic views about whether consciousness of given contents, such as perceptual objects and episodic memories, is graded (along a continuum from a fully unconscious grade to full conscious access) or rather all-or-none or dichotomous (i.e., either “black,” unconscious, or “white,” conscious). Another core opposition of perspectives is about whether consciousness (and in particular perceptual awareness) is temporally continuous in its flow, or rather discontinuous with a series of discrete conscious moments. A further one is about a view “from without” to conscious experiences (e.g., based on matching stimuli and responses) and a view “from within,” based on a first-person reflective access to conscious experiences across waking and dreaming. This article will consider how such apparently contrasting views can be reconciled in *synthetic perspectives*, also in light of recent experimental findings and developed theoretical perspectives.

Moreover, in cognitive psychology and neuroscience, as well as in psychology and neuroscience of emotion, the issue of consciousness appears crucial across multiple domains of investigations, such as perception, attention, cognitive control, memory, emotion, self, and decision making. Thus, consciousness research can play a crucial role for a unified understanding of cognitive processes, in terms of dynamic cognitive architectures, as well as for a deeper understanding of the interactions between cognition and emotion, and of the interactions of cognitive and emotional processes with the self. Such increased *third-person* insights about consciousness and related cognitive and affective processes based on scientific studies may interestingly converge with *first-person* insights about consciousness and mental (mind-body) processes gained through highly-trained (enhanced) attention and introspective skills in long-term mindfulness (insight) meditators (e.g., Lutz et al., 2008; Raffone and Srinivasan, 2009).

This article will in particular address current challenges about bridging consciousness with perception, cognition, self and emotion, with a focus on the following themes: (1) addressing the distinction between perceptual (phenomenal) awareness and conscious (cognitive) access; (2) highlighting reconciling views about long-standing controversies on the graded or all-or-none nature of consciousness, and (3) about the discrete vs. continuous process of consciousness; (4) discussing the views contrasting levels and a multidimensional approach to consciousness; (5) relating meta-awareness (metacognitive awareness) to earlier characterizations of consciousness; (6) reaching an increased understanding of the relationships between consciousness and aspects of the self; (7) developing a theoretical understanding of interoceptive awareness and mindfulness; and finally (8) developing a synthetic perspective beyond the opposition of the views “from within” and “from without” to conscious experiences, across waking and dreaming, with an epistemological balance, and with core implications for individual and collective well-being through the enhancement of human reflective awareness.

## Eight Grand Challenges in Consciousness Research

1. One of the main controversies in current consciousness research is about the distinction between *phenomenal consciousness* and *access consciousness*, advanced by the philosopher Ned Block (Block, 1995, 2005), which has led to remarkable scientific and philosophical discussions (Fazekas and Overgaard, 2018; Overgaard, 2018). Phenomenal consciousness refers to qualia, i.e., first-person experiences. Thus, *phenomenally* conscious content refers to subjective experience, such as in the perceptual experiences of red and green. *Access* conscious content is information which is “broadcast” in the conscious cognitive (global) workspace in the brain and made available to the brain's “consuming” systems, including systems for reasoning, planning, the evaluation of alternatives, decision making and the voluntary direction of attention. Block (1995, 2007) proposed that phenomenal content overflows cognitive access. Overgaard (2018) has recently argued that existing data do not demonstrate such overflow, and that, under the condition that cognitive access is defined as working memory or attention, overflow is theoretically possible, although highly difficult to empirically demonstrate. Naccache (2018) has recently observed five major problems raised by the distinction between phenomenal and access consciousness, based on a *global workspace* theoretical framework, that distinguishes between conscious access, which although limited in capacity at any given time enables access to widespread information sources in the brain in a global workspace system, and unconscious processing, involving information processing in a substantially segregated or modular fashion (Baars, 1997; Dehaene et al., 1998). It has also been remarked that recent research on working memory challenges the shared assumption that the representational capacity of cognitive access is fairly limited (Gross, 2018). However, the distinction between phenomenal and access consciousness and the overflow argument appear supported by transient vs. sustained recurrent mechanisms in the cerebral cortex (Lamme, 2003; Raffone and Pantani, 2010; Raffone et al., 2014), which are consistent with a global workspace theoretical framework, and can account for large sets of findings with attentional blink and visuo-spatial working memory paradigms (Simione et al., 2012). Moreover, attentional processes can operate at different stages, with differential influences on perceptual (phenomenal) consciousness and conscious (cognitive) access (Lamme, 2003; Raffone et al., 2014; Simione et al., 2020; Zivony and Eimer, 2020). Finally, the global workspace theoretical framework needs to take into account developments on *brain networks* (e.g., Bressler and Menon, 2010), that appear consistent with large-scale broadcasting in the brain before the stage of conscious access, which may support phenomenal consciousness. Further theoretical and empirical investigations appear needed on this challenging issue in consciousness research, including about understanding the roles of attentional processes in perceptual and access consciousness.
2. Recent developments in consciousness research include addressing whether consciousness is a *continuous* stream of percepts or it is *discrete*, i.e. occurring only at certain moments in time (e.g., VanRullen and Koch, 2003; Pöppel, 2009; Herzog et al., 2020). This issue has puzzled philosophers, psychologists, and neuroscientists for centuries. Herzog et al. (2020) have reviewed recent studies exploring long-lasting post-dictive effects, and, based on such review, have influentially proposed a two-stage discrete model in which substantial periods of continuous unconscious processing precede discrete conscious percepts. Herzog et al.'s model combines the advantages of both continuous and discrete models, and may contribute to resolve centuries old debates about perception and consciousness. The model emphasizes long-lasting unconscious processing, favoring global theories that have a two-stage architecture, such as global workspace models (Baars, 1997; Dehaene et al., 1998), and appear consistent with earlier theoretical proposals unifying attentional and consciousness theories (Raffone et al., 2014). Both slow evidence accumulation processes, in which widely distributed signals from several brain sources are gradually integrated, and discrete ignitions, with non-linear amplification processes leading to sharp transitions, can play a crucial role in such a model (Zylberberg et al., 2011; Raffone et al., 2014). Further studies appear needed to investigate the involved mechanisms, including electrophysiological and computational modeling investigations.
3. Another relevant issue in consciousness research is about the *dichotomous* (e.g., Sergent and Dehaene, 2004) vs. *graded* (e.g., Overgaard et al., 2006) nature of consciousness (conscious access). Recently, Sy et al. (2021) have found evidence that conscious perception can be both graded and discrete (all-or-none, dichotomous), by means of the attentional blink paradigm, in which two sequentially presented targets in a rapid serial visual presentation have to be detected. Their findings indicate that loss of target information can be graded or discrete, depending on whether perceptual or higher central stages are taxed by processing demands, and help reconcile conflicting views regarding how information can be lost from awareness. Such reconciling evidence resonates with earlier studies and theoretical proposals supporting the synthetic perspective that conscious perception can be both graded and discrete (Windey et al., 2013, 2014; Raffone et al., 2014). Windey et al. (2014), starting from a level of processing framework allowing for states of partial awareness, further elaborated their view that visual experience, as it is most often investigated in the literature, is both graded and all-or-none: low-level visual experience is graded, whereas high-level visual experience is all-or-none. The Theory of Attention and Consciousness (TAC) proposed by Raffone et al. (2014) can also be regarded as a synthesis of all-or-none and graded views of conscious representation. Indeed, through the stages of representations and processing in TAC both all-or-none ignition (sudden transition) processes, and gradual evidence accumulation, amplification and consolidation processes, take place. Further investigations appear needed to generalize the evidence across different experimental paradigms and to understand the implicated mechanisms. Moreover, there might be a relevant interdependence between the investigation of the issue of temporal continuity vs. discreteness of conscious processing and the issue of the graded vs. dichotomous nature of consciousness. Such interdependence might further extend to addressing the issue of perceptual (phenomenal) awareness as distinct from conscious (cognitive) access.
4. Several authors have agreed about the distinction between *contents* of consciousness, such as “the experience of redness,” “feelings of pain unpleasantness and intensity,” and “the taste of coffee,” and *levels* of consciousness, such as sleep, come, vegetative, and minimally conscious state, by also assuming distinct neural correlates of contents and levels of consciousness (Hohwy, 2009; Overgaard and Overgaard, 2010). However, Bayne et al. (2016) have challenged the notion of levels of consciousness, arguing that the levels-based framework for conceptualizing global states of consciousness is untenable, and have developed in its place a multidimensional account of global states. Bayne et al. argue that the task of identifying the dimensions of the space of global states of consciousness is an urgent and necessary one that will lead ultimately to a better understanding of consciousness itself. It can further be observed that there is a fundamental distinction between consciousness contents in terms of objects of perception and thought, and consciousness contents in terms of emotional and motivational states, which may rather be associated to global consciousness states, as in the refined ancient treatises of Buddhist psychology (Barendregt, 2006; Barendregt and Raffone, 2013; Raffone and Barendregt, 2020). This view can be associated to developments in affective neuroscience which emphasize the linking of emotional and cognitive parameters in mental states, with the implication of dynamic neural networks composed of interconnected pre-frontal and limbic brain structures (Salzman and Fusi, 2010). Future theoretical and experimental work is required to understand how these mental state representations form and how shifts between mental states occur, a critical feature of adaptive cognitive and emotional behavior, and to understand their relationships to global states of consciousness and processing of conscious contents.
5. Recent developments and challenges in consciousness research refer to *meta-awareness*, which can also be termed “*metacognitive awareness*” or “*reflective awareness*,” meant as the metacognitive function of being *reflectively* aware of the processes, contents and states of consciousness, including the processes and contents of conscious perceiving, thinking, and feeling (Schooler, 2002; Raffone and Srinivasan, 2009; Dunne et al., 2019). It has been suggested that the conscious processes, contents and states observed by meta-awareness are related to a first level of global workspace processing in the brain, whereas meta-awareness (metacognitive awareness) is linked to higher-order conscious processing and global workspace neurodynamics (Raffone and Srinivasan, 2009; Raffone and Barendregt, 2020). Meta-awareness has the potential to lead to deliberate changes in *mental programs*, i.e. conscious intentions or sequences of controlled processing steps in decision making and task performance, through cognitive monitoring, attentional control and other executive functions, which can be associated to transitions in the global workspace for conscious processing (Zylberberg et al., 2011; Barendregt and Raffone, 2013; Raffone and Barendregt, 2020). Meta-awareness has also been related to a fourth level of conscious processing (Raffone and Barendregt, 2020), beyond the unconscious, preconscious and conscious levels of contents in consciousness (Dehaene and Naccache, 2001). Meta-awareness has also been related to the notion of *mindfulness*, meant as a sustained and non-propositional form of meta-awareness (Barendregt and Raffone, 2013; Dunne et al., 2019). The issue of meta-awareness has been in particular linked to research on *mind wandering*, something referred to as task-unrelated thought, i.e., the experience of thoughts wandering even without intention and meta-awareness, with internally-directed attentional and working memory resources (Schooler, 2002; Seli et al., 2016). Given that conscious (cognitive) access resources or consumer systems (Block, 1995) may be mobilized in the absence of reflective awareness and intention in mind wandering, a challenge seems to be implicated for the notion of access consciousness, with a possible distinction between conscious *accessibility* (e.g., through thoughts) and *reflectivity* (e.g., through awareness of thoughts): cognitive access may take place without conscious reflectivity, i.e. absence of (meta)awareness of cognitive access itself, i.e., absence of reflectivity about the current allocation of attentional and working memory resources associated to conscious access, which would thus occur pre-reflectively.
6. It also appears crucial understanding the relationships between consciousness and self. Indeed, a fundamental aspect of conscious experiences is given by the experience of the *self* , i.e., the ongoing feeling that there is a unique entity or agent experiencing the world as a source of continuity, intentionality and identity. James (1892) characterized the self as “partly known and partly knower, partly object and partly subject” (p. 159), as the phenomenological correlate of unified mental life. James (1892) suggested that reflections on the self-need to address the fundamental distinction between an “*I*” and a “*Me*.” For James, the “I” is “that which at any given moment is conscious, whereas the Me is only one of the things which it is conscious of” (ibid., p. 175, italics in the original). The “I” or “knower” thus entails being the subject of experience, in contrast to the “Me,” as the object of experience or the “empirical aggregate of things objectively known” (ibid., p. 191). Authors from different theoretical perspectives have concurred with James on the distinction between an “I” corresponding to a subjective sense of the self as a thinker, causal agent and knower, and the “Me,” i.e., as the objective or explicit sense of the self with the unique and identifiable features constituting one's self-image or self-concept (e.g., Mead, 1934; Piaget, 1954; Gallagher, 2000). Currently, it appears particularly challenging to understand the relationships between contents (at different levels of access), consciousness levels or dimensions, phenomenal and access consciousness (see above), and different aspects of the self. These aspects of the self-include the *narrative self* (Gallagher, 2000), *autobiographical self* (Damasio, 1999), and *conceptual self* (Conway, 2005), which are linked to a *third-person perspective* related to the personal pronoun “Me.” They also include the *minimal self*, which, in contrast to the narrative self, was characterized by Gallagher (2000) as a pre-reflective aspect of the self, corresponding to the personal pronoun “I,” which is increasingly investigated in philosophical, psychological, neuroscientific and clinical studies (e.g., Lane, 2020). The minimal self refers to a sense of being the immediate subject of experience in the present and to taking on a *first-person perspective*. The minimal self is characterized as involving a very basic pre-reflective self-consciousness. Phenomenologists explain this as a structural feature of the flow of consciousness implicated with its intrinsic temporality, and with the involvement of *retention* and *protention* processes at any moment (Gallagher and Zahavi, 2008). Retention may be related to working memory, and protention to *predictive processing* (Friston, 2009), the latter being the subject of a number of theoretical and empirical investigations in cognitive neuroscience, also of relevance for research on consciousness and the self (Timmermans et al., 2012). The minimal self can be associated to a momentary mode of self-awareness, which has been found to be enhanced by mindfulness training, whereas the influence of the narrative self-decreases with mindfulness training (Farb et al., 2007; see also Tagini and Raffone, 2010). An important challenge to address is whether consciousness can take place in the absence of any form of self, as suggested by research on deep meditation states, through an enhanced mindful (reflective) awareness (Dor-Ziderman et al., 2013).
7. Moreover, a challenging research issue of current relevance for conscious research is developing an increased understanding of *interoception* and *interoceptive awareness*, as also related to other domains of conscious contents. Interoception is the perception of sensations arising from inside the body, which include sensations related to internal organ function such as heart beat, respiration, satiety, as well as the autonomic nervous system activity related to emotions (Cameron, 2001; Craig, 2002). Much of these interoceptive perceptions remain unconscious, while information that becomes conscious, i.e., in interoceptive awareness, becomes available to conscious awareness (Cameron, 2001). Interoceptive awareness can be trained by *mindfulness meditation*, with increased emotion regulation skills (Price and Hooven, 2018), thus also with relevance for psychological well-being and psychopathology. Theoretically, interoceptive conscious awareness can be characterized in terms of different levels of contents in consciousness, as in other domains of conscious contents (Dehaene and Naccache, 2001; Raffone and Barendregt, 2020), and can also be interestingly included in the debates about perceptual (phenomenal) and access consciousness (i.e., in terms of the distinction between interoceptive phenomenal and access consciousness), as well as about the bodily, experiential and affective elements of the self (Gallagher, 2013), and their relationships with pre-reflective and reflective processes, including thoughts (e.g., related to cognitive appraisal and reappraisal) and mindful awareness.
8. Finally, a further challenge is balancing a perspective “*from within*” with a perspective “*from without*” on conscious experiences. A perspective from within can be linked to the phenomena of *dreaming* and *lucid dreaming*, as related to core issues in consciousness research (Revonsuo, 2006), such as about the distinction between phenomenal consciousness and access consciousness (Fazekas and Nemeth, 2018; Pantani et al., 2018), and the relationships with aspects of the self (Pantani et al., 2018). Indeed, dream experiences involve images, emotions and thoughts in complex and integrated scenes with a narrative structure within which the dreamer is immersed; these scenes can include intense interactions with animate and inanimate objects, as well as complex storylines (e.g., Limosani et al., 2011). Revonsuo (2006) interestingly observed that although dreaming and waking consciousness differ with respect to the causal paths of their production, they are ontologically equivalent; moreover, since consciousness itself can be regarded as a process of simulation, not only are dreams experiences but, in a way, all experiences are dreams (p. 55). This approach ‘from within' to consciousness and its neural correlates, especially with the involvement of participants trained in introspection, such as in meditative states and lucid dreaming, might shed important light on consciousness processes, including on the causal paths of production of conscious experiences, as well as on the entanglement of perceptual, cognitive, affective and self-elements, and meta-awareness. This perspective on consciousness “from within” across waking and dreaming may appear in contrast with approaches “from without” to consciousness that emphasize reportability and matching of conscious experiences with external stimuli (e.g., Dehaene et al., 2006). From this latter point of view, perceptual contents are conscious only if they can be reported and correctly matched to presented stimuli. In another synthetic perspective, Pantani et al. (2018) argued that these apparently contrasting (“from without” and “from within”) approaches to conscious experiences can be reconciled by revealing unified processes, structures and mechanisms across waking and dreaming, with different causal paths of production for conscious experiences in awake perception and dreams, within a unitary phenomenological and neurocognitive framework. Finally, as related to metacognitive awareness of conscious experiences (including meta-awareness during mind wandering, moment-by-moment mindfulness in meditation and everyday life, and lucid dreaming), the same causal paths of production of *reflective awareness* in conscious experiences (see above) can cut across all conscious experiences. Understanding this reflective or *higher-order awareness*, which can be potentially lit up with all conscious experiences across waking and dreaming, including meditative states and all activities of everyday life, can indeed be regarded as one of the greatest challenges for consciousness research, also with outstanding implications for individual and collective well-being. Indeed, such human reflective awareness can be effective to lead to changes in individual and collective views and habits, also with core ethical implications. Thus, developments in consciousness research, with an epistemological balance of first- and third-person perspectives, can have a great impact on our society and world.

## Conclusion

The grand challenges in consciousness research addressed in this article, among other relevant ones, show the relevance and dynamicity of this field of knowledge and scientific investigation, as also related to other areas of psychology and neuroscience, among other implicated disciplines. Such challenges interestingly interact with each other, and can be addressed through synthetic perspectives beyond oppositions of views. An overarching reflectivity of human awareness with and beyond percepts, dreams, feelings, desires, values, and thoughts appears implicated across such challenges, also with relevant implications for well-being and ethics, thus also interacting with crucial challenges that humanity is facing in these times.

Further relevant epistemological and methodological challenges implicated in consciousness research need to be carefully addressed, with the importance for experiments to question given theories, rather than merely attempting to support them. In this respect, Yaron et al. (2021) have considered a large set of studies that interpreted their findings with reference to as least one of four leading neuroscientific theories of consciousness. They found that supporting a specific theory can be predicted solely from methodological choices, irrespective of findings, and that, furthermore, most studies interpreted their findings *post-hoc*, rather than a-priori testing critical predictions of the theories.

## Author Contributions

The author confirms being the sole contributor of this work and has approved it for publication.

## Conflict of Interest

The author declares that the research was conducted in the absence of any commercial or financial relationships that could be construed as a potential conflict of interest.

## Publisher's Note

All claims expressed in this article are solely those of the authors and do not necessarily represent those of their affiliated organizations, or those of the publisher, the editors and the reviewers. Any product that may be evaluated in this article, or claim that may be made by its manufacturer, is not guaranteed or endorsed by the publisher.
